# Mild poikilocapnic hypoxia increases very low frequency haemoglobin oxygenation oscillations in prefrontal cortex

**DOI:** 10.1186/s40659-021-00362-2

**Published:** 2021-12-14

**Authors:** Agnieszka Gruszecka, Monika Waskow, Marta A. Malkiewicz, J. Patrick Neary, Jyotpal Singh, Taylor Teckchandani, Gregory P. Kratzig, Magdalena Wszedybyl-Winklewska, Andrzej F. Frydrychowski, Jacek Rumiński, Natalia Głowacka, Piotr Lass, Pawel J. Winklewski, Marcin Gruszecki

**Affiliations:** 1 Department of Radiology Informatics and Statistics, Medical University of Gdansk, Tuwima Str. 15, 80-210, Gdansk, Poland; 2 Institute of Health Sciences, Pomeranian University of Slupsk, Slupsk, Poland; 3 Department of Human Physiology, Applied Cognitive Neuroscience Lab, Medical University of Gdansk, Gdansk, Poland; 4 Department of Psychiatry, Medical University of Gdansk, Gdansk, Poland; 5 Faculty of Kinesiology and Health Studies, University of Regina, Regina, Canada; 6 Department of Psychology, University of Regina, Regina, SK Canada; 7 Department of Human Physiology, Medical University of Gdansk, Gdansk, Poland; 8 NIRTI SA, Wroclaw, Poland; 9 Department of Biomedical Engineering, Faculty of Electronics, Telecommunications and Informatics, Gdansk University of Technology, Gdansk, Poland; 10 Department of Nuclear Medicine, Medical University of Gdansk, Gdansk, Poland

**Keywords:** Hypoxia, Wavelet transform, Blood pressure, Subarachnoid space width, Near infrared spectroscopy

## Abstract

**Background:**

The aim of the study was to investigate the effect of mild cerebral hypoxia on haemoglobin oxygenation (HbO_2_), cerebrospinal fluid dynamics and cardiovascular physiology. To achieve this goal, four signals were recorded simultaneously: blood pressure, heart rate / electrocardiogram, HbO_2_ from right hemisphere and changes of subarachnoid space (SAS) width from left hemisphere. Signals were registered from 30 healthy, young participants (2 females and 28 males, body mass index = 24.5 ± 2.3 kg/m^2^, age 30.8 ± 13.4 years).

**Results:**

We analysed the recorded signals using wavelet transform and phase coherence. We demonstrated for the first time that in healthy subjects exposed to mild poikilokapnic hypoxia there were increases in very low frequency HbO_2_ oscillations (< 0.052 Hz) in prefrontal cortex. Additionally, SAS fluctuation diminished in the whole frequency range which could be explained by brain oedema.

**Conclusions:**

Consequently the study provides insight into mechanisms governing brain response to a mild hypoxic challenge. Our study supports the notion that HbO_2_ and SAS width monitoring might be beneficial for patients with acute lung disease.

## Introduction

Hypoxia is frequently observed in a number of clinical situations such as lung disease, sleep apnoea, as well as cardiac and cerebrovascular dysfunction associated with aging. Mild hypoxia is also an eminent feature of the respiratory distress syndrome evoked by the coronavirus (SARS-CoV-2): severe acute respiratory syndrome (SARS). A number of cognitive and neuropsychiatric manifestations of SARS have been observed in a proportion of patients such as depressed mood, anxiety, post-traumatic stress disorder and cognitive decline. Although these are multifactorial, changes in cerebral oxygen supply and related pathophysiological consequences may play a role [1].

It is well-known that hypoxia leads to a decline in haemoglobin oxygenation (HbO_2_) in cerebral cortex [2]. Diminished oxygen supply to the brain results in several compensatory mechanisms, which in turn trigger substantial changes in various aspects of brain functioning. Augmented cerebral blood flow [3] and cerebral blood volume [4, 5] together with blood–brain barrier impairment [6] lead to discrete brain oedema [4, 5] and increases in intracranial pressure [4, 5].

Brain hypoxia also results in a number of metabolic changes associated with an augmented cerebral metabolic rate of oxygen, lactate and glutamate concentrations accompanied by a diminished creatinine concentration. These metabolic alterations suggest higher neural activity and increased oxidative metabolism [7, 8]. Despite these adaptations cytotoxic cerebral oedema may develop and subsequently evolve into ionic cerebral oedema, thus increasing grey matter volume even if blood–brain barrier still remains intact [9].

Transcranial Doppler studies demonstrate that acute mild normobaric hypoxia (15% O_2_) augments both blood pressure (BP) and cerebral blood flow velocity (measured in middle cerebral artery) oscillations in the very-low-frequency range (0.02–0.07 Hz), while does not affect BP and cerebral blood flow velocity fluctuations at cardiac and respiratory frequencies. These changes (at very low frequency), associated with increase in coherence and transfer function gain were typically explained by weakened autoregulatory control [10, 11].

The autoregulation dogma is however constantly challenged with increasing emphasis on either local neural or metabolic mechanisms [12, 13]. The role of intracranial pressure in the feedback loops regulating widely understood cerebral metabolic homeostasis is continuously highlighted [13, 14]. Consequently, in this study we aimed at assessing the effect of hypoxia on the oscillatory behaviour of BP, oxygenated haemoglobin (HbO_2_), electrocardiogram (HR/ECG), and subarachnoid space (SAS) width using established non-invasive methods [15, 16].

The relative concentrations of the oxygenated and deoxygenated haemoglobin can be measured in the prefrontal area with near infrared spectroscopy (NIRS) [15]. Oscillatory changes in the SAS width, reflecting cerebrospinal fluid pulsatility [16], can be instantly monitored with the method developed by our team, called near-infrared transillumination-backscattering sounding (NIR-T/BSS) [16–19]. Both methods can be used simultaneously [20]. Wavelet transform analysis of biological signals can elegantly quantify and delineate the investigated interactions in both frequency and time domains [12, 21]. The method is ideal to analyse oscillations of biological signals and was used many times by different groups.

Another useful mathematical tool to analyse biological signals are coupling functions which describe very precisely any interactions between considered systems. The coupling functions reveal information about the coupling strength but also are helpful to find the mechanism responsible for considered interactions. They determine the possibility of qualitative transitions between the oscillations e.g. routes into, and out of, phase synchronization. They are very suitable for studying interaction of oscillatory processes in physiology e.g., cardiorespiratory or cardiovascular interactions [22, 23]. Recently, Gruszecka et al. [24] studied the respiratory–cardiac coupling of the blood pressure and cerebrospinal fluid signals. Another interesting study was carried out by [25]. Combined wavelet transform and coupling functions helped to consider an interactions between three oscillations: respiratory, cardiac and vascular activity. The oscillations were extracted from three different signals: respiratory effort signal, electrocardiogram and laser Doppler flowmetry. Another example is that general anaesthesia can lead to important changes in the forms of coupling function between brain waves [26]. The detailed review about coupling functions and its application can be found in Stankovski et al. [27].

We hypothesized that mild hypoxia would increase the prominence of very low-frequency HbO_2_ local brain oscillations while maintaining the impact of central oscillatory components generated by the heart and lungs. We also expected SAS oscillations to diminish in the whole frequency range as a result of simulated altitude at approximately 2900–3000 m.

## Results

Four signals were continuously and simultaneously recorded from the volunteers: BP, HR/ECG, HbO_2 RIGHT_ and SAS_LEFT_ while breathing normal room air (19.8% O_2_), and during normobaric hypoxia (14.8% O_2_) in an environmental chamber to simulate altitude at 2900–3000 m. The atmospheric pressure for whole time of experiment was kept at the same level 712 mmHg, while the level of oxygen during normobaric hypoxia was reduced to 14.8%. A gas mixture of 19.8% O_2_ was used as the normoxic condition as this is the native partial pressure of oxygen in Regina, Saskatchewan, Canada (577 m above sea level) where the testing was performed. According to nonparametric Wilcoxon rank sum test (see Table 1) there was a statistically significant change of all measured signals during mild normobaric hypoxia.

**Table 1:** Subject characteristics during breathing a gas mixture with 19.8% and 14.8% O_2_

	Gas mixture with 19.8% O_2_	Gas mixture with 14.8% O_2_
HR (beats/min)	$60.87_{68.41}^{54.34}$	$66.41_{75.75}^{57.38}$^***^
DBP (mmHg)	$65.45_{70.74}^{59.55}$	$69.36_{74.26}^{62.56}$^*^
SBP (mmHg)	$113.98_{120.18}^{103.98}$	$119.63_{129.35}^{110.63}$^***^
MAP (mmHg)	$82.09_{88.35}^{74.77}$	$87.51_{91.84}^{79.91}$^*^
SAS_LEFT_ (AU)	$0.19_{0.25}^{0.15}$	$0.17_{0.23}^{0.14}$^*^
HbO_2 RIGHT_ (%O_2_)	$0.71_{0.87}^{0.54}$	$0.52_{0.56}^{0.36}$^***^
tHb (µM)	$9.28_{11.36}^{7.55}$	$14.76_{16.64}^{13.02}$^**^
SaO_2_ (%O_2_)	$98_{99}^{97}$	$92_{94}^{91}$^***^
EtCO_2_ (mmHg)	$33.11_{35.17}^{31.63}$	$31.59_{33.75}^{30.05}$^***^
EtO_2_ (mmHg)	$127.65_{130.43}^{124.54}$	$96.52_{99.78}^{94.25}$^***^

Values shown are ${\text{median}}_{{75{\text{th percentiles}}}}^{{25{\text{th percentiles}}}}$. p values were estimated between two stages of the experimental procedure

*HR* heart rate; *DBP* diastolic blood pressure; *SBP* systolic blood pressure; *MAP* mean arterial pressure, *SAS*_*LEFT*_ subarachnoid width; *HbO*_*2 RIGHT*_ relative changes in oxyhaemoglobin; *tHb *relative changes in total haemoglobin; *SaO*_*2*_ oxyhaemoglobin saturation; *EtCO*_*2*_ end-tidal CO_2_; *EtO*_*2*_ end-tidal O_2_

*p  < 0.05; **p  < 0.01; ***p  < 0.001

**Fig. 1:**
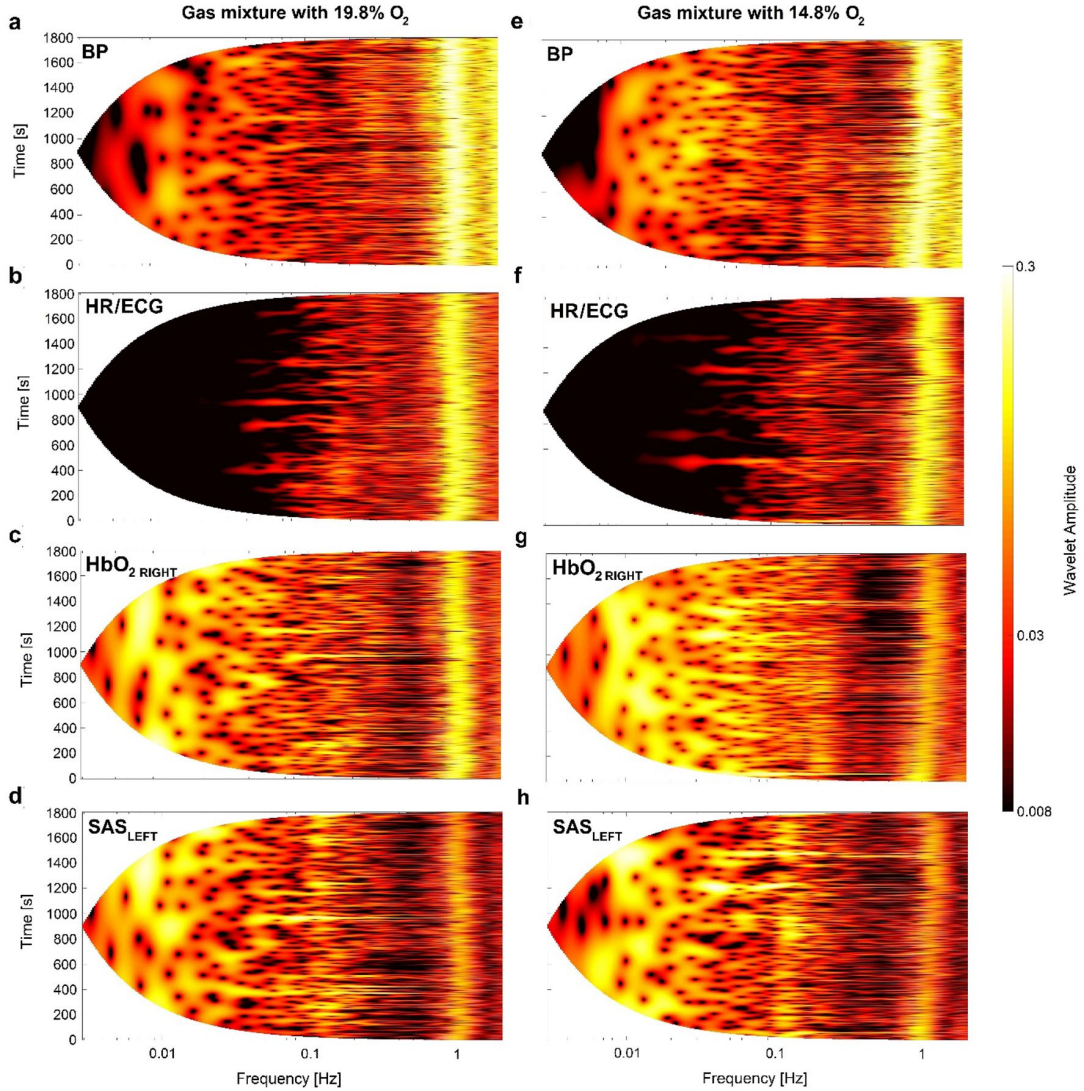
Wavelet transform of recorded signals: BP (**a**, **e**), HR/ECG (**b**, **f**), HbO_2 RIGHT_ (**c**, **g**) and SAS_LEFT_ (**d**, **h**) for one of the volunteers. Left (right) columns of plots illustrate results for breathing a gas mixture with 19.8% (14.8%) O_2_

Figure 1 shows the result of the amplitude of the wavelet transform (WT) for one representative volunteer. Left and right columns of Fig. 1 corresponds to breathing a gas mixture with 19.8% and 14.8% O_2_, respectively. We estimated WT for all four measured signals: BP (a, e), HR/ECG (b, f), HbO_2 RIGHT_ (c, g) and SAS_LEFT_ (d, h).

**Fig. 2:**
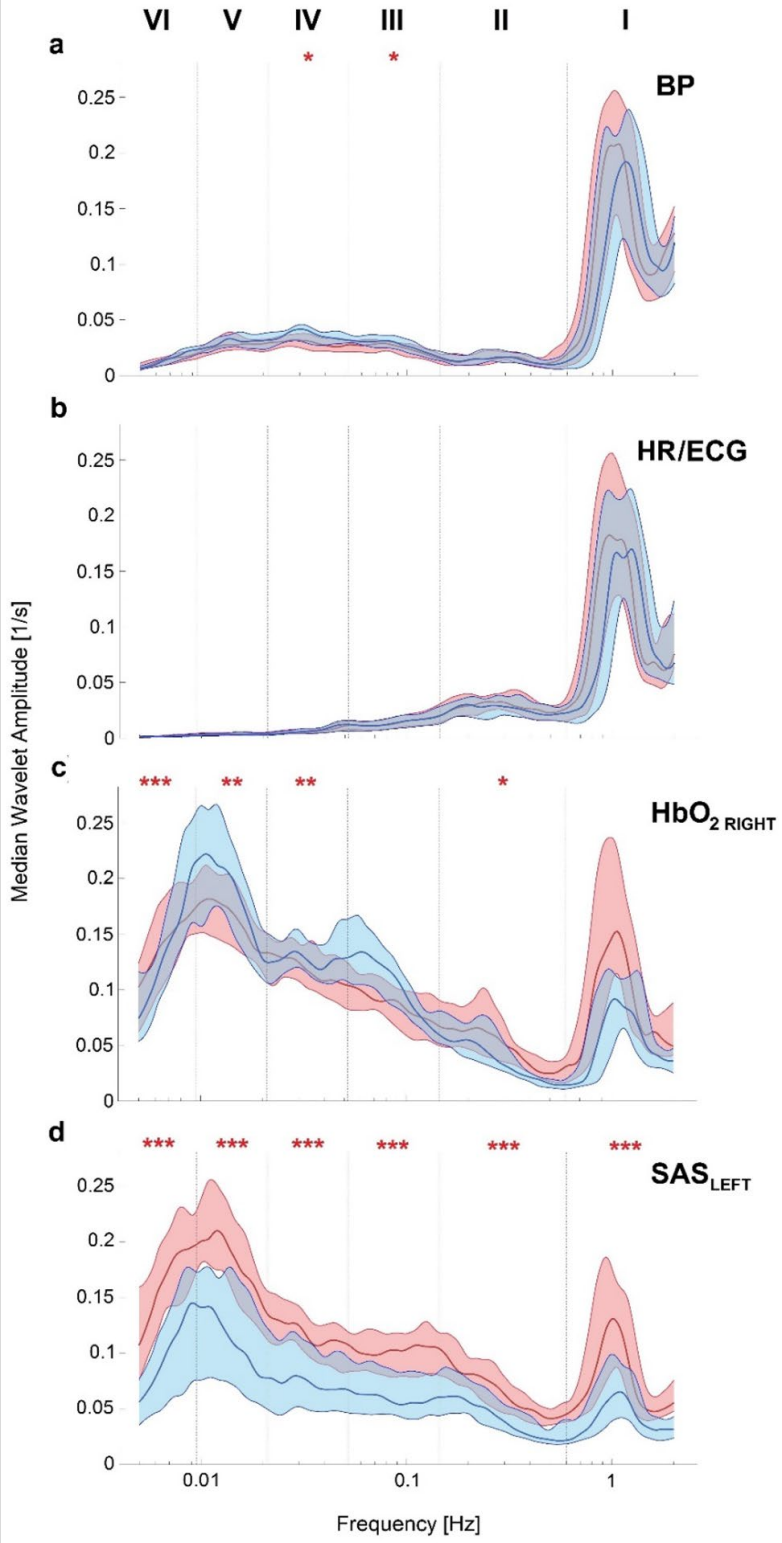
Median (thick lines) of the time-averaged wavelet transforms of signals recorded in all 30 subjects: **a** BP, **b** HR/ECG, **c** HbO_2 RIGHT_ and **d** SAS_LEFT_ obtained from the 30 min continuous recordings. Shaded areas indicate the inter-quartile range (25th, 75th percentiles). Red (blue) lines and shade areas correspond to breathing a gas mixture with 19.8% (14.8%) O_2_. *p  < 0.05; **p  < 0.01; ***p  < 0.001

To simplify the comparison between two stages of experimental procedure in terms of their frequency content, we plotted the median of the time-averaged amplitude of wavelet transforms (Fig. 2).

**Fig. 3:**
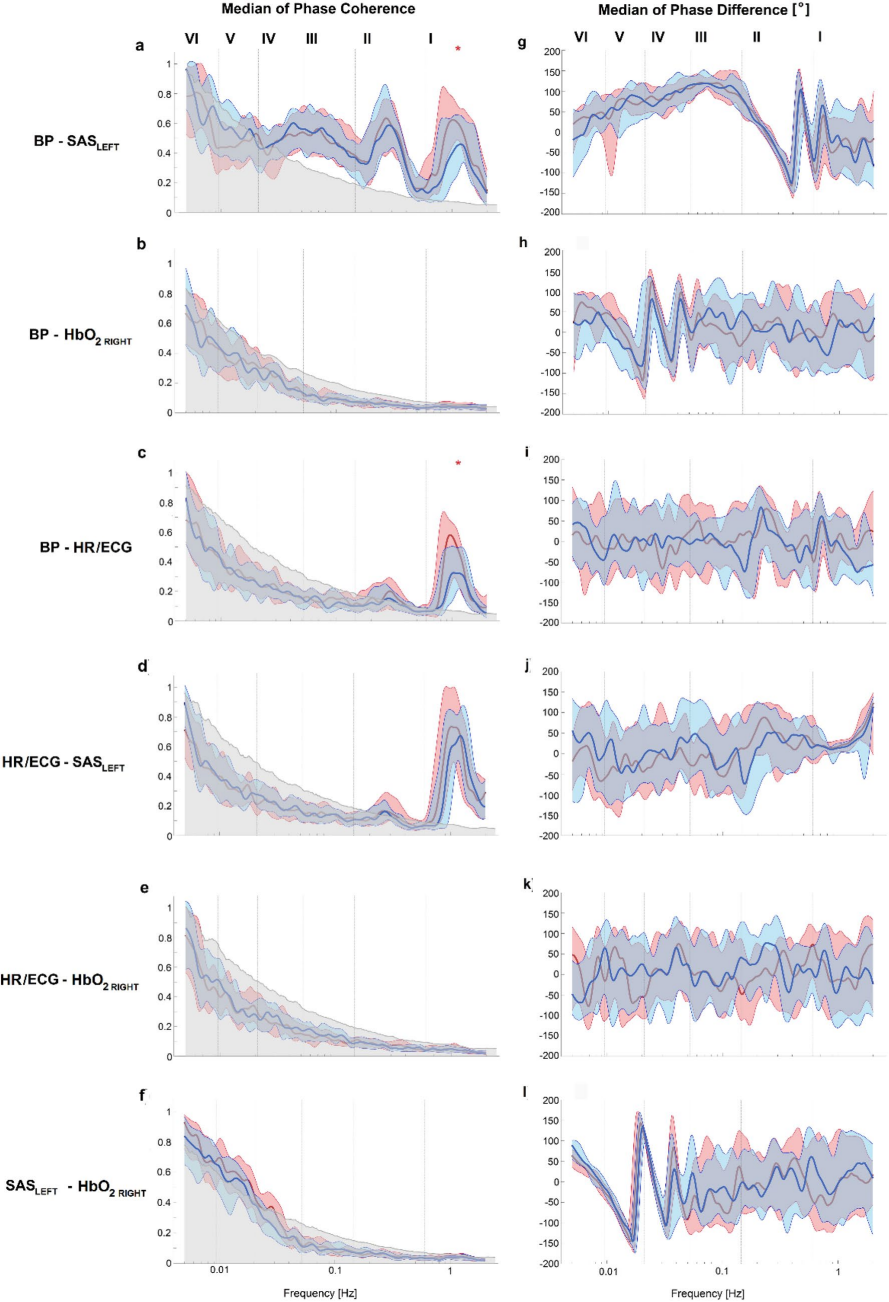
Median (thick lines) of wavelet phase coherence (left column) and phase difference (right column) between **a**, **g** BP vs SAS_LEFT_, **b**, **h** BP vs HbO_2_, **c**, **i** BP vs HR/ECG, **d**, **j** HR/ECG vs SAS_LEFT_, **e**, **k** HR/ECG vs HbO_2_ and **f**, **l** SAS_LEFT_ vs HbO_2_. Coloured shading indicates the interquartile range (25th, 75th percentiles) for 30 subjects. Coherence below the 95th percentile of the surrogates (light grey line and shading) is not considered significant. Red (blue) lines and shade areas correspond to breathing a gas mixture with 19.8% (14.8%) O_2_. *p  < 0.05; **p  < 0.01; ***p  < 0.001

Figure 3 illustrates the wavelet phase coherence (left column) and phase difference (right column) between pairs of all collected signals. The value of phase coherence was significant when the value of phase coherence was higher than 95th percentile of 435 (2-permutation of 30 subjects) inter-subject surrogate. When we observed statistically significant phase coherence at certain frequencies, we assumed the same for phase differences. For phase coherence we found statistically significant differences for BP-SAS_LEFT_ and BP-HR/ECG only for the cardiac interval. We did not observe any statistically significant difference for phase difference for all considered pairs of signals.

## Discussion

The novel findings of our study showed that mild poikilocapnic hypoxia increases the very low frequency HbO_2_ oscillations (< 0.052 Hz) in prefrontal cortex while diminishing SAS oscillations in the whole frequency range. In addition we confirmed that prefrontal cortex HbO_2_ and SAS width decline during exposure to lowered oxygen partial pressure.

In recent years there has been an increased interest in cerebrovascular mechanisms related to normal and pathological physiology. As such, we have witnessed a substantial paradigm shift in our thinking in regard to brain perfusion control mechanisms. The increasing accumulation of evidence suggests that astrocytes act as physiological sensors reacting to changes in the brain parenchymal levels of metabolic substrates (such as oxygen), cerebral perfusion and intracranial pressure changes [13, 28–30]. In particular, astrocytes might be responsible for local cellular signalling mechanisms adjusting cerebral perfusion to brain metabolic needs [13].

Our study strongly supports the view that local brain mechanisms, rather than systemic control-level mechanisms are responsible for adaptation to mild poikilocapnic (normoxic) hypoxia. We observed decreases in respiratory contribution to HbO_2_ prefrontal cortex oscillations, while at the same time observing substantially increased very low frequency HbO_2_ oscillations (< 0.052 Hz). Very low frequency periodic dynamism is believed to reflect neural sympathetic and metabolic activity [12, 31], and is most likely linked to increased neural metabolism [7].

Prefrontal cortex HbO_2_ fluctuations at the 0.145–0.052 Hz frequency were reported to be linked to general BP oscillations [32–34]. In response to normobaric hypoxia, BP fluctuations did not change in the 0.145–0.052 frequency. We also did not observe any phase difference in the HbO_2_ and BP signals. Stabilisation of BP-brain signals oscillations at the 0.145–0.052 Hz frequency are most likely driven by increased sympathetic activity related to hypoxia and slightly increased intracranial pressure [4, 5, 35]. We have previously shown that high sympathetic drive strengthen the links between BP and SAS signals [14].

Slightly increased intracranial pressure and discrete brain swelling previously reported in hypoxia could have resulted in a decline of SAS oscillations across the whole frequency range. Although we cannot confirm discrete brain swelling from our study, our findings extend previous results collected in our laboratory where healthy subjects were exposed to 5 min of poikilokapnic (normobaric) hypoxia, and we observed a trend towards a decline in SAS width and diminished BP-SAS amplitude coupling [36]. In the current study, we observed diminished BP-SAS and HR/ECG-BP phase coherences within the cardiac frequency band. Suppression of respiratory SAS oscillations were reported previously by our team as an early indicator of rising intracranial pressure in rabbit [18]. A decline in the modulation of cerebral blood flow by even moderately increased intracranial pressure was also observed in humans [37].

This study used poikilocapnic (normobaric) hypoxia to challenge whether EtCO_2_ fluctuations varied in response to reflexive hyperventilation. We preferred this over an isocapnic hypoxic challenge, defined by hypoxia in the presence of maintained CO_2_ within the bloodstream, as we believe our chosen methodology better mimicked clinical conditions. A gas mixture of 19.8% O_2_ was used as the normoxic condition as this is the native partial pressure of oxygen in Regina, Saskatchewan, Canada (577 m above sea level) where the testing was performed.

Our study demonstrates that clinical situations associated with even mild cerebral hypoxia may result in several pathophysiological adaptations including changes in brain metabolism and cerebrospinal fluid dynamics. Non-invasive modalities that enable continuous brain monitoring could provide several benefits to patients suffering from diseases associated with declined brain oxygenation, stroke or traumatic brain injury.

### Conclusions

In subjects exposed to mild poikilokapnic hypoxia we have demonstrated for the first time that: (1) very low HbO_2_ oscillations (< 0.052 Hz) increase in prefrontal cortex, and (2) SAS fluctuation diminishes in the whole frequency range. Consequently the study provides insights into the physiological mechanisms governing brain response to a mild hypoxic challenge. Our study supports the notion that HbO_2_ and SAS width monitoring might be beneficial for patients with acute lung disease.

## Materials and methods

### Subjects

A group of 30 healthy and not smoking volunteers (28 males and 2 females, age 30.8 ± 13.4 years, BMI  = 24.5 ± 2.3 kg/m^2^) were involved in the experiments, which were carried out in accordance with the recommendations of Helsinki. The Ethics Committee of University of Regina (REB#2017-013) approved this study and the experimental protocol. All subjects were older than 18 years and signed a consent form to participate in the study. Participants were asked to avoid any alcohol at least 24 h before the experiment, and tea, coffee, nicotine, cocoa and any food and beverages containing methylxanthine for at least 12 h before the experiment. Intense exercise training was not allowed at least 6 h prior to testing, and all subjects were asked to void their bladder within 30 min of testing. All procedures were preceded by 10 min of rest in the sitting position in a comfortable chair located in quiet room.

### Experimental design

Two tests of breathing a gas mixture with 19.8% and 14.8% O_2_ were conducted in a quiet room and environmental chamber. Room and chamber were pre-set to a temperature of 18–20 °C. After all of the medical research equipment was attached to the volunteer participant (see “Measurements” Section below), they were instructed to lie supine on a bed with a pillow to support their head during breathing a gas mixture of normal room air (19.8% O_2_) and during normobaric hypoxia (14.8% O_2_). The first 30 min of experiment, subjects were asked to breath room air (19.8% O_2_), and then they moved to the environmental chamber with the aid of a research assistant (it took about 3–5 min). During the next 30 min, volunteer subjects breathed freely in the hypoxic chamber with reduced levels of oxygen (14.8%). The hypoxic oxygen condition mimiced the elevation of approximately 2900–3000 m above sea level.

### Measurements

A Finometer (Finapres Medical Systems, Arnhem, The Netherlands) was used to measure BP. Finger photoplethysmography BP was calibrated against brachial arterial pressure. The signal was collected from the left middle finger at all times during both normoxia (19.8% O_2_) and hypoxia (14.8% O_2_). HR was determined from an ECG signal. The ECG ground electrode was placed on the left anterior superior iliac spine and the two main leads under the middle portion of each clavicle (Lead I) [20]. SaO_2_ was measured using a Nellcor PM10N Portable SpO_2_ Patient Monitoring System (Medtronic Canada, Vancouver, BC). During the experiment, the device was placed on the right index finger. Expired respiratory gas samples from the mouthpiece were constantly analysed using the side-stream technique for EtCO_2_ using AD Instruments ML206 gas analyser (Colorado Springs, USA). SAS signal was collected using an SAS Monitor (NIRTI SA, Wierzbice, Poland). A detailed description of the SAS Monitor has been provided previously (e.g., [16]. The NIRS signal was collected by PortaLite system (Artinis Medical, The Netherlands). The sensor was positioned over pre-frontal cortex of the right hemisphere during the experiment. NIRS contains transmitters at 30, 35 and 40 mm from the receiver, which allows a penetration depth of approximately one-third to one-half of the distance between optodes [38]. During the experiment, we collected the following NIRS signals: relative changes in oxy-(HbO_2_), deoxy-(HHb), total haemoglobin (tHb  =  HbO_2_  +  HHb) and haemoglobin difference (Hb_diff_  =  HbO_2_ − HHb). To avoid admission of background light the NIRS sensor was secured with a black coloured tensor bandage. We decided to analyse a NIRS signal that penetrate the deepest regions of head to be sure that we truly analyse signals from brain vessels. Like many others, [39–42] we focused our analyses on the oxyhaemoglobin (HbO_2_) signal. Additionally, to avoid any interference between NIRS and SAS signals, we recorded only SAS_LEFT_ from left hemisphere and HbO_2 RIGHT_ from right hemisphere.

All parameters were recorded and saved simultaneously for further analysis. This was completed for both segments, with the first 30 min normoxic condition completed first, followed by the subsequent 30 min normobaric hypoxic second. To import and view collected signals, a PowerLab 8/32 amplifier was coupled with LabChart 7 Pro (AD Instruments, Colorado Springs, Colorado, USA). All signals before analysis were down sampled to 10 Hz, detrended using a moving average with a window size of 220 s, and normalized by subtraction of their mean and division by their standard deviation.

### Wavelet transform

To detect and perform analysis of physiological processes that are responsible for generating oscillations in the cardiovascular system, we used wavelet analysis. The wavelet transform is a method that transforms a signal from the time domain to the time–frequency domain. The definition of the wavelet transform is:

$$W\left( {s,t} \right) = \frac{1}{\sqrt s }\mathop \smallint \limits_{ - \infty }^{ + \infty } \varphi \left( {\frac{u - t}{s}} \right)g\left( u \right)du,$$

where $W\left( {s,\;t} \right)$ is the wavelet coefficient, $g\left( u \right)$ is the time series and $\varphi$ is the Morlet mother wavelet, scaled by factor $s$ and translated in time by $t$. The Morlet mother wavelet is defined by the equation:

$$\varphi \left( u \right) = \frac{1}{{\sqrt[4]{\pi }}}{\text{exp}}\left( { - i2\pi u} \right){\text{exp}}\left( { - 0.5u^{2} } \right)$$

where $i = \sqrt { - 1}$. The rationale for using the Morlet wavelet is that it affords good localization of events in time and frequency due to its Gaussian shape [39, 42]. The wavelet coefficients are complex numbers in the time–frequency plane when the Morlet wavelet is used:

$$X\left( {\omega_{k} ,\;t_{n} } \right) = X_{k,n} = a_{k,n} + ib_{k,n} .$$

They define the instantaneous relative phase,

$$\theta_{k,n} = {\text{arctan}}\left( {\frac{{b_{k,n} }}{{a_{k,n} }}} \right),$$

and the absolute amplitude,

$$\left| {X_{k,n} } \right| = \sqrt {a_{k,n}^{2} + b_{k,n}^{2} } ,$$

for each frequency and time.

During the measurement, hypoxia may create phase modulations. A mathematical tool to find the relationship between the phases of two signals is the wavelet phase coherence (WPCO). WPCO enables us to determine whether the oscillations detected are significantly correlated over time. To estimate the WPCO we used the following expression [43]:

$$C_{\theta } \left( {f_{k} } \right) = \frac{1}{n}\left| {\mathop \sum \limits_{t = 1}^{n} {\text{exp}}\left[ {i\left( {\theta_{2k,n} - \theta_{1k,n} } \right)} \right]} \right|,$$

where $\theta_{k,n} = {\text{arctan}}\left( {\frac{{b_{k,n} }}{{a_{k,n} }}} \right)$ is an instantaneous measure of phases at each time ${\text{t}}_{{\text{n}}}$ and frequency $f_{k}$ for both signals. When two oscillations are unrelated (related), their phase difference continuously changes (remain constant) with time, thus their $C_{\theta } \left( {f_{k} } \right)$ approaches zero (one).

Additionally, we can calculate the phase difference $\Delta \theta_{k}$, which provides information about the phase lag of one oscillator compared to the other:

$$\Delta \theta_{k} = \arctan \left( {\frac{{\frac{1}{n}\mathop \sum \nolimits_{t = 1}^{n} \sin \left( {\Delta \theta_{2k,n} - \Delta \theta_{1k,n} } \right)}}{{\frac{1}{n}\mathop \sum \nolimits_{t = 1}^{n} \cos \left( {\Delta \theta_{2k,n} - \Delta \theta_{1k,n} } \right)}}} \right),$$

$$where \Delta \theta_{k} \in \left( { - 180^\circ , 180^\circ } \right).$$

### Statistical analysis

To avoid the assumption of normality in the results, nonparametric statistical tests were used for all comparisons. The Wilcoxon rank sum test was used to compare whether the median of results for breathing a gas mixture with 19.8% O_2_ and 14.8% O_2_ was significantly different. The results of our calculations are found in Table 1; Figs. 2, 3.

Our analysis were supported by added six different frequency intervals which correspond to different physiological functions described previously by Stefanovska et al. [21] and Gruszecki et al. [16] (see Figs. 2, 3). Additionally, we estimated the p value of the differences between the results for breathing a gas mixture with 19.8% and 14.8% O_2_ for all measured signals. We observed the highest differences (p  < 0.001) for WT amplitude for SAS_LEFT_ for all frequency intervals. For HbO_2 RIGHT_ the differences between amplitude of wavelet transform were associated with respiration (II, 0.145–0.6 Hz), neurogenic (IV, 0.021–0.052 Hz), endothelial nitric oxide (NO) dependent (V, 0.0095–0.021) and NO independent (VI, 0.005–0.0095 Hz). In turn, WT amplitude of the BP signal only has differences (p  < 0.05) in myogenic (III, 0.052–0.145 Hz) and neurogenic (IV) intervals. We did not observe any statistically significant differences for the HR/ECG signal.

To test whether the estimated values of phase coherence are statistically significant, the surrogate data testing method was used [44]. As we know there are naturally less cycles of oscillations the lower in frequency that we consider. This can cause artificially increased wavelet phase coherence at low frequencies, even in cases where there is none. The surrogate analysis helps us to find a significance level above which the phase coherence may be regarded as physically meaningful. To estimate significance level, we used intersubject surrogates [45], which assumes that the signals collected from different subjects must be independent, while having similar characteristic properties**.** For 30 volunteers we used 435 intersubject surrogates. The actual value of phase coherence obtained at each frequency can then be compared with the surrogate threshold. When the phase coherence is located above the threshold it is considered to be statistically significant.

## Data Availability

The data presented in this study are available on request from the corresponding author. The data are not publicly available due to privacy.

## Abbreviations

HbO_2_: Haemoglobin oxygenation
BP: Blood pressure
SAS: Subarachnoid space
WT: Wavelet transform
NIRS: Near infrared spectroscopy
HR: Heart rate
DBP: Diastolic blood pressure
SBP: Systolic blood pressure
MAP: Mean arterial pressure
tHb: Relative changes in total haemoglobin
O_2_: Oxygen
SaO_2_: Oxyhaemoglobin saturation
EtCO_2_: End-tidal CO_2_
EtO_2_: End-tidal O_2_
NO: Nitric oxide
WPCO: Wavelet phase coherence
